# Obesity population at risk of COVID-19 complications

**DOI:** 10.1017/gheg.2020.6

**Published:** 2020-11-06

**Authors:** Sarah Cuschieri, Stephan Grech

**Affiliations:** 1 Department of Anatomy, Faculty of Medicine and Surgery, University of Malta, Msida, Malta; 2 Department of Trauma and Orthopaedics, Mater Dei Hospital, Msida, Malta

**Keywords:** Coronavirus, epidemic, Global Health, obesity, pandemic, Population Health

## Abstract

**Introduction:**

Global public health is challenged by two concurrent epidemics; COVID-19 and obesity. Considering the global prevalence of obesity, exploring relationships with COVID-19 are of clinical importance. The aim was to provide a comprehensive summary and recommendations on this relationship between COVID-19 and obesity.

**Method:**

A literature search was performed to prepare a narrative review of COVID-19 and obesity.

**Results:**

An obesity state promotes chronic inflammation, vitamin D deficiency, hinders immunity and causes mechanical lung compression. These increase susceptibilities to COVID-19 infection, complications including the requirement of invasive ventilation. Existing co-morbidities enhances these complications. Preventive measures of social distancing and self-isolation may increase stigmatisation and psychological deterrents. Hence, special recommendations targeting this vulnerable population are required.

**Conclusion:**

The obese population is a COVID-19 vulnerable group, requiring special attention during this pandemic to avoid complications and healthcare systems burden. Lacking COVID-19 vaccination, regular physical activity and a healthy diet are recommended with attention to mental health. A prolonged quarantine duration and administration of prophylactic vitamin D may be considered.

## Introduction

The global public health body is being challenged by two concurrent epidemics, SARS-CoV2, also known as COVID-19 and obesity. SARS-CoV2 is an influenza-like virus that has affected millions of people across the globe [1]. A link between influenza and obesity was reported during the 2009 Influenza A virus H1N1 pandemic. At the time, individuals with obesity had an increased risk of getting a severe disease as well as increase in hospitalisation and mortality rates [2]. Similarly, an association has been identified between overweight and obese status and severity of COVID-19 infection [3]. Unfortunately, a large proportion of the world's population has been reported to be either overweight or obese, hence increasing the risk of acquiring a severe COVID-19 infection [4]. This indirectly will increase the burden on the healthcare systems with a possibility of collapse [5]. Therefore, it is considered timely to explore the relationship between COVID-19 and obesity and their clinical importance. In this review, we aimed to provide a comprehensive summary of the literature and recommendations on the relationship between COVID-19 and obesity.

## Method

A literature search using ‘COVID-19’, ‘Obesity’ and ‘Abdominal Obesity’ as the keywords was performed through PubMed and Google search engines. Published articles pertaining to the aim of this review were considered. Furthermore, the search was extended to international organisations websites in order to establish recommendations and information published by these entities for the population with obesity. The international organisations included were: World Health Organization (WHO), Centres for Disease Control and Prevention (CDC), World Obesity and European Association for the Study of Obesity.

## Obesity, inflammatory, thrombotic responses and COVID-19

Obesity is defined as a body mass index (BMI) higher than 30 Kg/m^2^ [6]. Those individuals with severe obesity, defined as BMI of 40 Kg/m^2^ or higher have been labelled as ‘vulnerable’ by the ‘Centres for Disease Control and Prevention’ (CDC) [7]. While the WHO together with the World Obesity Federation have declared that both overweight and obesity are potential risk factors for worse COVID-19 outcomes [8,9]. In fact, it has been reported that the majority of COVID-19 patients admitted to the intensive care unit (ICU) were obese [10].

The obesity phenotype consists of the accumulation of adipose tissue, which is responsible for the development of localised inflammation and promotion of chronic systemic low-grade inflammation [11]. The pro-inflammatory markers C-reactive protein and interleukin-6 are elevated as a response to the excess adipose mass. Similarly, the pro-inflammatory product tumour necrosis factor and the anti-fibrinolytic adipokine levels are also increased. On the other hand, the anti-inflammatory adipokine, adiponectin, is suppressed. Hence, in the presence of obesity, there is a metabolic dysfunction resulting from an imbalance in the expression of pro- and anti-inflammatory adipokines [12]. Subsequent to the suppression of adiponectin, macrophages modulation is affected [12]. Macrophages infiltrate the adipose tissue leading to inflammation, hypoxia, augmentation of chemokine and adipocyte cell death. The adaptive immune system is activated with the accumulation of macrophages within the inflamed adipose tissue. CD8 + effector T cells are responsible for a phenotypic switch of the macrophages to the proinflammatory M1 macrophage phenotype, which has a role in the development of insulin resistance. There is also an accumulation of NK cells that are described as innate-like T lymphocytes which have a role in adipose tissue inflammation and glucose intolerance [13]. This chronic low-grade inflammatory response leads to the impairment of the immune response and chemotaxis with a subsequent disturbance of the immune surveillance system [14]. Local respiratory inflammatory responses also occur through the increase in mast cells causing susceptibility to airway disease [15]. Severe forms of COVID-19 are characterised by acute systemic inflammation responses with high circulatory levels of proinflammatory cytokines levels that lead to multiorgan failure [16]. Hence, in light of the obesity pathophysiological low-grade systemic inflammation and the cytokine storm caused by severe forms of COVID-19, an obese individual acquiring COVID-19 is at a very high risk of an escalated systemic inflammation with potentially fatal outcomes. This systemic inflammation can have a deterrent effect on the blood flow within arteries precipitating plaque rupture and resulting in pulmonary embolus, stroke or myocardial infarction. In fact, in a Dutch study, it was reported that 31% of the ICU COVID-19 admissions had venous or arterial thrombotic complications [17].

COVID-19 infection has also been found to be linked with coagulation disturbances and thrombotic mechanisms. It was reported that complement-mediated microvascular injury was identified in both the skin and lung post-mortem. Deposits of C5b-9, C4d and MASP2 within the microvasculature of these two systems indicated the activation of both the lectin-based and alternative pathways [18]. Furthermore, elevated D-dimer and fibrin degradation product levels were established in patients with COVID-19 pneumonia [19,20]. Therefore, considering that both COVID-19 infection and obesity pathophysiology are associated with coagulation disturbances, individuals with obesity are at higher risk of thrombotic events in the eventuality of a COVID-19 infection.

## Obesity, COVID-19 and pulmonary disease

The increase in abdominal circumference in obesity compresses the lung structure while putting tethering forces between the lung parenchyma and the airway. Reduced oxygen saturation at the lung bases with impaired ventilation has been associated with abdominal obesity [15]. The respiratory system has angiotensin-converting enzyme 2 (ACE2) receptors which are responsible for anti-inflammatory responses. The enveloped spike glycoprotein of the SARS-CoV-2 enters human cells, including the pulmonary system, through ACE2 [21]. It was postulated that in severe COVID-19 infections there may be an imbalance in the ACE2 activation pathways with an increase in the pro-inflammatory response [22]. The SARS-CoV-2 interaction with the renin-angiotensin-aldosterone system may lead to a severe hypokalaemic state with increased susceptibility to tachyarrhythmias. Furthermore, hypokalaemia increases the vascular permeability with a potential risk of respiratory distress syndrome. Therefore, COVID-19 infected obese individuals are at an additional risk of an exaggerated systemic inflammatory and electrolyte imbalance that may have a potentially fatal outcome.

Published reports from the COVID-19 pandemic experiences state that a substantial proportion of individuals with obesity required hospitalisation due to COVID-19 with a high mortality rate [23,24]. It appears that obese patients younger than 60 years are mostly susceptible to such admissions [25]. Furthermore, individuals with obesity experienced a worse COVID-19 respiratory outcome requiring ICU admissions for invasive ventilation [26]. This increase in intensive care brings forward other hardships due to potential increased difficulty in intubation, central intravenous access, as well as most ICUs’ beds, are not designed to accommodate a high body weight [27]. Moreover, due to weight and girth limits associated with CT-scans and MRIs, which are the main diagnostic imaging modalities used for infections including COVID-19, a proportion of the obese population would be hindered from undergoing such investigations [27]. These factors may hinder the essential care required by this vulnerable population during the pandemic.

If the H1N1 influenza is considered, it was reported that an increase in interleukin-8 is released in obese individuals when compared to lean individuals [28]. Furthermore, on investigating the effect of the influenza vaccine on obesity, it was found that the CD8 + T-cell responses were defective when compared to the healthy weight population while the levels of influenza vaccine antibody declined. This increases the risk of acquiring a severe influenza disease in the obese population due to the inability to mount an immune response [29]. Therefore, considering the already weakened immune response of the obese population, the presence of localised pulmonary inflammation and the obese response to the H1N1 influenza virus, it is expected that a deterrent effect would be present if COVID-19 infection is acquired. Furthermore, the delayed innate and adaptive immune responses in the obese allow for an increase in viral replication, spread and prolonged infections [30]. Additionally, the loss of leptin receptors leads to an impaired viral clearance possibly arising from immune cell-mediated interactions within the respiratory epithelium [31]. Hence, it may be postulated that a similar viral effect can occur in a COVID-19 infected obese individual.

## Obesity and other co-morbidities

It is a well-known fact that an individual with obesity may have other underlying co-morbidities such as type 2 diabetes and cardiovascular disease [32–34]. Individuals with either disease have also been declared as being vulnerable to COVID-19 infection [35]. The presence of multiple concurrent chronic diseases contributes to multimorbidity, a state that has been linked to higher COVID-19 infection risk, worse health outcomes and increased mortality [36]. This comes about from the ability of SARS-CoV-2, as already stated, to access the host cells via the ACE2. The ACE2 is not only found in the lungs but is also expressed by endothelial cells [37]. Therefore, COVID-19 can affect a number of different organs.

An impaired cell-mediated immune response forms part of the type 2 diabetes pathophysiology, putting these individuals at higher risk of acquiring infections [38,39]. If infected by COVID-19, the virus enters through the ACE2 receptor found within pancreatic beta cells [21]. An acute onset of hyperglycaemia occurs due to beta-cell dysfunction [40]. Therefore, individuals with obesity and type 2 diabetes are at a higher risk of complications if infected by COVID-19. This may explain the higher rates of ICU admissions by those who are obese.

It is not uncommon that individuals with obesity also have underlying cardiovascular co-morbidities. Cardiovascular complications have been reported in COVID-19 infected individuals through various modes. Direct myocardial injury can occur through a similar pathway as in diabetes, where the SARS-CoV-2 enters the myocardial cells through ACE2. The ACE2 has a role in the neurohumoral regulation of the cardiovascular system. Binding of the coronavirus leads to an alteration in the ACE2 signalling pathways resulting in acute myocardial injury [41]. The overall incidence of a COVID-19 acute myocardial injury has been reported to vary between 8% and 12% [42,43]. Furthermore, in the presence of a systemic infection, there is a higher cardiometabolic demand along with hypoxia arising from acute respiratory distress that will impair the myocardial oxygen demand–supply relationship leading to an acute myocardial injury [44]. It has been reported that a substantial proportion of COVID-19 patients requiring hospitalisation had cardio-cerebrovascular disease and hypertension with an associated three-fold and two-fold increased risk of ICU admissions [42].

## Obesity, vitamin D and COVID-19

Vitamin D has an effect on the modulation of both the innate and the adaptive immune responses [45]. A deficiency of this fat-soluble vitamin D in individuals with obesity has been extensively reported [46–48]. This may result from multifactorial factors including insufficient exposure to solar radiation [49], inadequate intake of vitamin D rich food [50], decrease in liver production of 25-hydroxy vitamin D due to liver steatosis [51] and reduction in the bioavailability of this vitamin [52]. It is not uncommon that obese individuals have hyperparathyroidism, that also causes secondary vitamin D deficiency [53]. Hence, integrating the pathophysiology of obesity with an altered immune response and the fact that these same individuals can also be vitamin D deficient, the susceptibility to infection, including COVID-19 is enhanced. Recent communications have suggested that vitamin D deficiency enhances COVID-19 infection susceptibility while it was also suggested that vitamin D should be given as a prophylaxis to all acutely ill patients with COVID-19 [54,55]. Further research is recommended to explore the relationship between vitamin D and COVID-19.

## Obesity and stigma

Stigma towards individuals with an obese bodily shape is common. This causes numerous negative consequences effecting their mental and physical health [56]. The preventive measures imposed by governments to contain the COVID-19 spread may have a psychological toll on individuals with obesity. In individuals who are already stigmatised and experiencing higher rates of depression, the social distancing and self-isolation measures might tip the balance [27].

There is a higher chance that these individuals will consume processed and tinned food rather than fresh food, to avoid going out to the supermarket. The COVID-19 lock-down and self-isolation may further enhance such habits. Another factor is the actual physical space available to exercise at home. Considering that gyms and other exercise facilities have been asked to shut down as part of the COVID-19 containment measures, a true problem of physical inactivity is further compounded. Hence, it is important that a media campaign using different modes of delivery targeting this vulnerable group is set up to promote a healthy choice of food as well as to engage in physical activity that can be easily done at home. A number of entities have set up physical activity workouts over social media to engage all the population to be physically fit. One can also observe that some TV stations are hosting a daily physical activity regimen for those watching. Other initiatives have been set up including various helplines even targeted to aid the mental health during the COVID-19 pandemic [57].

## Recommendations for obesity

A safe and effective COVID-19 vaccine is still being worked on by the pharmaceutical companies, hence it is paramount that other measures are instituted to protect the population especially the vulnerable groups. Protecting the vulnerable forms one of the seven principles for COVID-19 ethics along with population health maximisation and solidarity [58]. The WHO and the World Obesity Federation have issued a number of recommendations targeted at governments to ensure that national strategies are set up to safeguard the short- and long-term health of the population [8]. Although the containment measures of social distancing, staying at home and banning of public gatherings are paramount during this pandemic, it is essential that governments, clinicians and individuals alike, acknowledge the impact these measures will have on the population's health. Lockdowns and fear of contracting the virus can lead to reliance on high fat, sugar and salted processed long-life foods with a reduction in consumption of fresh fruit, vegetables and unprocessed meat. This dietary change increases the risk of weight gain, especially in the absence of physical activity opportunities when staying at home. Such a scenario will have a deterrent effect on those already struggling with overweight or obesity. Additionally, there is a risk of previously normal-weight individuals to progress to an overweight or obese status during this pandemic. In fact, this issue is a major concern for the World Obesity Federation and the WHO [8,9]. This further enhances the potential presence of food insecurity that the vulnerable population might experience due to reduced access to shops and food assistance programmes [8]. It was recommended that governments should ensure adequate availability of nutritious food supply especially to those with pre-existing health conditions as well as the vulnerable groups. The provision of nutritious food is only one side of the coin. Physical activity is another very important requisite for healthy living. With the implementation of the government's preventive measurements, opportunities for physical activity have been restricted to the home. Even on easing of some restrictions, most recreational places remained closed to avoid mass gatherings. Of note, physical activity has a dual effect of weight loss and immune-modulatory actions. Undergoing regular physical activity enhances cytokines production mediated through the toll-like receptors signalling pathways that improve host resistance towards pathogen invasion [59]. Furthermore, physical activity enhances the antioxidant defence system as well as the oxidative stress reduction [60]. Hence, encouraging physical activity should be on every government's agenda.

Recommendations were put forward for the implementation of policies that allow individuals to undergo physical activity in open spaces while maintaining the required social distancing [8]. Social and other types of media are good tools to disseminate the appropriate advocacy on the importance of the physical activity to the nation [8]. A number of different organisations and sports entities have set up online videos with different physical activity routines that are easily done at home. Some countries have also engaged national television stations in broadcasting daily exercise routines for their viewers to follow. Obviously, it must be advised that such exercise programmes should be tailored to the individual's capacity to exercise. The media has a critical role in disseminating clear and transparent guidance on COVID-19 as well as providing information about helplines for vulnerable groups among others.

It is important to note that during this difficult time, individuals are more psychologically vulnerable, and this may impinge on their mental health. Isolation, lack of social engagement, changes in the daily routine and reduced physical activity are all triggers that may impact on mental health. Vulnerable groups including the obese population are more at risk of such mental health impact. This may be coupled up with the potential distress arising from the compromised treatment access that people living with obesity may be facing, such as cancellations of follow-up consultations with physicians and reductions in bariatric surgery during this pandemic. Hence, it was recommended that governments recognise the mental health impact that COVID-19 has on the population and provide supporting services through virtual means as well as helplines [8]. Furthermore, COVID-19 response strategies targeting the non-communicable disease population including obesity have been recommended. These should ensure adequate prevention, management, support and advice to the vulnerable population and their caregivers [9]. During this pandemic, a number of physicians have shifted their practice to telemedicine in order to continuously support their patients while abiding by social distancing. Vulnerable groups including those living with obesity should be encouraged to use such virtual means, both for management and for psychological help. Here, again the media should play an important role in disseminating information about such services.

Epidemiological data have established that the incubation period of COVID-19 can last up to 14 days. However, in the previous influenza pandemic, obese individuals were found to have a prolonged shedding period [61]. Considering such evidence, international organisations may consider recommending a prolonged quarantine (presumably doubled) for people living with obesity with respect to lean individuals. Furthermore, considering the established COVID-19 pathophysiological coagulopathy risk, prophylactic anti-coagulants may be recommended for those with obesity requiring hospitalisation. Equally, regular electrolyte balance monitoring is required during the hospitalisation duration. Nonetheless, Vitamin D prophylaxis should be considered as being implemented as a recommendation for people living with obesity especially following the containment strategies to stay at home.

## Conclusion

Individuals with obesity are part of the vulnerable groups for COVID-19. Mechanical respiratory restriction and metabolic inflammatory responses resulting from the increased adiposity hinders the respiratory system. Hindrance of both innate and adoptive immune response increases the susceptibility for severe infections. This may enhance the need for invasive ventilation in ICUs upon acquiring COVID-19. The presence of other co-morbidities especially type 2 diabetes and cardiovascular diseases further aggravate the COVID-19 infection. Preventive COVID-19 measures should also incorporate the encouragement for healthy fresh food consumption and physical activity due to their positive immune modulation. A number of COVID-19 response strategies recommendations have been put forward by international organisations to ensure prevention, management and safety of the population health. A prolonged quarantine duration and administration of prophylaxis vitamin D may be considered as part of the preventive strategies for individuals with obesity.
